# Enhancing the quality of life for palliative care cancer patients in Indonesia through family caregivers: a pilot study of basic skills training

**DOI:** 10.1186/s12904-016-0178-4

**Published:** 2017-01-17

**Authors:** Martina Sinta Kristanti, Sri Setiyarini, Christantie Effendy

**Affiliations:** School of Nursing, Faculty of Medicine, Universitas Gadjah Mada, Yogyakarta, Indonesia

**Keywords:** Indonesia, Basic skills training, Palliative care, Quality of life, Cancer

## Abstract

**Background:**

Palliative care in Indonesia is problematic because of cultural and socio-economic factors. Family in Indonesia is an integral part of caregiving process in inpatient and outpatient settings. However, most families are not adequately prepared to deliver basic care for their sick family member. This research is a pilot project aiming to evaluate how basic skills training (BST) given to family caregivers could enhance the quality of life (QoL) of palliative care cancer patients in Indonesia.

**Methods:**

The study is a prospective quantitative with pre and post-test design. Thirty family caregivers of cancer patients were trained in basic skills including showering, washing hair, assisting for fecal and urinary elimination and oral care, as well as feeding at bedside. Patients’ QoL were measured at baseline and 4 weeks after training using EORTC QLQ C30. Hypothesis testing was done using related samples Wilcoxon Signed Rank. A paired t-test and one-way ANOVA were used to check in which subgroups was the intervention more significant.

**Results:**

The intervention showed a significant change in patients’ global health status/QoL, emotional and social functioning, pain, fatigue, dyspnea, insomnia, appetite loss, constipation and financial hardship of the patients. Male patient’s had a significant effect on global health status (qol) (*p* = 0.030); female patients had a significant effect on dyspnea (*p* = 0.050) and constipation (*p* = 0.038). Younger patients had a significant effect in global health status/QoL (*p* = 0.002). Patients between 45 and 54 years old had significant effect on financial issue (*p* = 0.039). Caregivers between 45 and 54 years old had significant effect on patients’ dyspnea (*p* = 0.031).

**Conclusions:**

Basic skills training for family caregivers provided some changes in some aspects of QoL of palliative cancer patients. The intervention showed promises in maintaining the QoL of cancer patients considering socio-economic and cultural challenges in the provision of palliative care in Indonesia.

## Background

Chronic non-communicable diseases such as cancer pose a major, ongoing public health problem and are responsible for 60% of deaths in Southeast Asia [1]. These conditions are also strongly correlated with poverty. Surveillance of these diseases and their risk factors needs to be improved, and health care systems must be strengthened to address the needs of the patients through primary health care and appropriate referral systems [1].

Similar to other Southeast Asian countries, Indonesia faces a range of challenges in providing health services for the poor. These include unavailability and unaffordability of services, as well as lack of adequate staff, transportation, and equipment [2]. Many patients in Indonesia tend to postpone cancer treatment because of their lack of knowledge on treatment options, insufficient financial resources, the side effects of treatment, and the paternalistic approach of health professionals [3]. The survival rate for cancer patients in Indonesia is resultantly lower compared with that of other Southeast Asian countries [4, 5].

Although a universal health insurance system has been in place in the country since 2005, it has not worked as well as planned [6, 7], and economically disadvantaged patients still typically have to rely on family members to care for them rather than seeking professional treatment [8]. Unsurprisingly, wealthier patients generally tend to have better treatment outcomes than financially disadvantaged ones [9].

In Indonesia, strong family bonds underpin the high degree of familial involvement during patients’ care and hospitalization [10]. Nurses assume important roles in activities pertaining to daily living, physical, spiritual, social, psychosocial, autonomous, and financial aspects during periods of hospitalization. It has also been reported that physicians primarily focus on physical symptoms, and that supporting individuals such as social workers and volunteers play a largely insubstantial role [10]. At home, the family provides the majority of care, mostly with no expert support from visiting nurses, physiotherapists, or occupational therapists.

Indonesia still has a lack of formal institutions to support patients with long-term conditions, as seen in the absence of hospices and respite care. Therefore, disadvantaged patients with terminal illness are typically cared for at home by family members [8] who generally have little or no training in provision of basic care. The lack of necessary knowledge and skills may cause family caregivers to have a lack confidence and feelings of uncertainty, which can lead to unease and anxiety [11]. Family caregivers of patients with advanced stage cancer may have similar experiences [12]. Basic skills training (BST) for family caregivers is therefore crucial.

### Aim of study

The research question underlying the present pilot study pertained to the effects that BST for family caregivers had on cancer patients’ quality of life (QoL). Using two-tailed testing, an intervention was tested to determine how BST affect the QoL of palliative cancer patients.

## Methods

### Participants

Head nurses in selected cancer wards in the major teaching hospital in Yogyakarta identified patients who could potentially serve as research participants. Inclusion criteria were: (i) stage 3 or 4 cancer, (ii) Palliative Performance Scale (PPS) score of less than 60, (iii) patients and family caregivers consented to participating in the study, and (iv) family caregivers were functionally literate. Following an examination of medical charts, 41 potential participants and their family caregivers were found to meet the study inclusion criteria and agreed to participate. Of these, 30 continued for the duration of the study.

### Outcome measurement

The major outcome variable, QoL, was assessed using the European Organization for Research and Treatment Cancer Quality of Life C30 version 3 (EORTC QOQL C-30) [13, 14]. This instrument is used worldwide and has been translated into more than 60 languages, including Indonesian [15]. The EORTC QLQ-30 consists of 30 questions divided into three subscales: global health status/QoL, functional scales, and symptom/single items. There are only two questions on global health status/QoL scale. The functional scale consists of five subscales: physical (PF), role (RF), emotional (EF), cognitive (CF), and social (SF). Symptom/single items consist of nine discrete variables: fatigue (FA), nausea/vomiting (NV), pain (PA), dyspnea (DY), insomnia/sleep (SL), appetite loss (AP), constipation (CO), diarrhea (DI), and financial hardship related to illness (FI) [13].

The functional and symptom/single subscales are measured on a four-point Likert scale, from 1 (“not at all”) to 4 (“very much”). An exception is for the global health status/QoL scale, which is based on a seven-point Likert scale, from 1 (“very poor”) to 7 (“excellent”) [13]. For Functional and Global health status/QoL, a higher score indicates a better QoL. In contrast, a higher score for symptoms and single items indicates poorer QoL [14].

### Intervention

We planned an intervention centered on a BST educational package that we developed to encourage interaction between family caregivers and nurse educators [11, 16]. The package included a 1-h video (on CD) and a module consisting of five chapters on assisting a bedridden patient with bathing, providing oral hygiene, hair washing, assisting with urination and bowel movements, and managing feeding orally and using a nasogastric tube. Nurse educators demonstrated these skills and provided assistance sessions to family caregivers for practicing their skills on patients. The nurse educators were given a 1-day training in advance about the research objectives and procedures, measurement of research outcomes, and patient education theory.

### Data collection

**Fig. 1:**
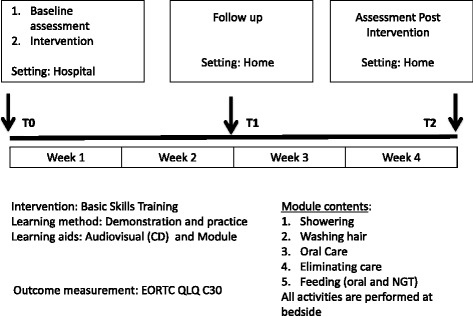
Time line and intervention details

Three training sessions for the family caregivers were provided (Fig. 1). The first was held at the hospital 1 week prior to the patient’s discharge. The family caregivers observed an initial demonstration by the nurse educators, watched the video, and then practiced the skills. Prior to this, the selected patients had provided signed informed consent, and baseline data on the patients’ QoL were collected.

Two weeks later, the nurse educators visited individual patients’ homes to give encouragement to the family caregivers and assist them in developing their skills in providing personal care, and to provide further information if required. On the third visit, within 1 month after patients’ discharge from the hospital, nurse educators visited the patients’ homes and collected further data on the patients’ QoL. An interval of 2 weeks after the second visit was chosen as the most suitable time between the follow up visits because it was reported in a similar study that 39.3% of cancer patients in a palliative condition passed away within 3 weeks [17].

### Analysis

Data were cleaned and assessed for normality, descriptive analysis was performed using frequency and mean to analyze patient demographics and QoL. The paired data testing was conducted using related samples with Wilcoxon Signed Rank tes. Cohen’s *d* test was used to calculate the effect size; scores of 0.2, 0.3, and 0.8 were considered small, moderate, and high, respectively [16]. A paired t-test and one-way analysis of variance (ANOVA) were used to investigate in which subgroups an intervention might be effective. Statistical significance was set at *p* < 0.05.

## Results

Of the 41 participants who entered the study, one decided not to continue, three could not be reached for contact after the second visit, and seven died before the program was evaluated. This left 30 participants who completed the study; their results are reported herein (Table 1).

**Table 1:** Demographics data

Characteristics	Patient (*n* = 30)	Caregiver (*n* = 30)
Sex, *n* (%)		
Male	8 (27)	12 (40)
Female	22 (73)	18 (60)
Age in years		
18 - 44	9 (30)	18 (60)
45 - 54	10 (33)	8 (27)
> 55	11 (37)	4 (13)
Type of Cancer		
Breast	9 (30)	
Digestive (colon, recti, sigmoid)	5 (17)	
Gynaecology (Vulva, Ovarian, Cervical)	5 (17)	
Non Hodgkin Lymphoma	4 (13)	
Head and Neck	4 (13)	
Osteosarcoma	2 (7)	
Thyroid	1 (3)	
Time since diagnoses (in months)		
1 to 3	7 (23)	
3 to 5	5 (17)	
> 5	18 (60)	
Income		
< 100 USD/month	19 (63)	
100 – 300 USD/month	8 (27)	
> 300 USD/month	3 (10)	
Palliative Performance Score (PPS)		
< 30 (Poor functioning)	7 (23)	
40 – 60 (Moderate functioning)	23 (77)	
Treatment		
Chemotherapy only	10 (33)	
Radiotherapy only	1 (3)	3
Both chemotherapy & radiotherapy	5 (17)	17
None	14 (47)	47
Education background		
Elementary school	9 (30)	5 (17)
Junior high school	6 (20)	4 (13)
Senior high school	11 (37)	16 (53)
University	4 (13)	5 (17)
Relationship with patient		
Spouse		15 (50)
Non-spouse		15 (50)
Live with patient		
Yes		24 (80)
No		6 (20)
Status of employment		
Work		15 (50)
Unemployed		15 (50)
Experience in caregiving		
Yes		12 (40)
No		18 (60)

### Characteristic of participants

As shown in Table 1, the ratio of female to male participants was around 3:1. Most patients were above 55 years old (37%) and approximately one-third were diagnosed with breast cancer. Most participants were not newly diagnosed, as they reported having been informed more than 5 months prior. Over 60% of the participants had a monthly income of less than USD 100. Ten of 30 received chemotherapy only and most received neither chemotherapy nor radiotherapy (47%). A closer inspection revealed that 77% had moderate functional ability based on their PPS score, while 23% had poor functional ability. Fifty percent of participants were being cared for by their spouses and 60% of the family caregivers were female. Sixty percent of family caregivers were under 44 years old, 80% lived with the patients, over half had graduated from senior high school, and 60% had no previous caregiving experience.

### Quality of life

In general, scores for almost all items in the EORTC QLQ C30 increased after intervention. As shown in Table 2, participants’ global health status/QoL ratings significantly improved after intervention from *M =* 40.27; SD = 17.79 to *M* = 56.94; SD = 18.05 with *p* = 0.001. For the functional scales, EF and SF improved after intervention, with some positive changes in PF, CF, and RF. Those last three items did not improve significantly after intervention (*p* = 0.225, 0.418, and 0.431, respectively). As for symptoms and single items, there were significant reductions in FA (*p* = 0.022), PA (*p* = 0.028), DY (*p* = 0.02), SL (*p* = 0.013), AP (*p* = 0.030), CO (*p* = 0.004), and FI (*p* = 0.009). The other symptoms, such as nausea and diarrhea, showed declining trends, but were not statistically significant (*p* = 0.243 and 0.097, respectively). The majority of effect sizes were small to medium, at 0.10–0.53, except for global health status/QoL, at 0.92.

**Table 2:** QOL score (EORTC QLQ C-30) pre and post intervention (*n* = 30)

Subscale	Mean score		Effect size	*P*-value
	Pre (SD)	Post (SD)		
Global health status/QOL^a^	40.27 (17.79)	56.94 (18.05)	0.92	0.001
Functional Scales^b^				
Physical (PF)	11.98 (15.98)	17.11 (22/8)	0.26	0.225
Role (RF)	11.11 (19.24)	13.33 (21.62)	0.10	0.418
Emotional (EF)	63.33 (30.21)	79.44 (26.77)	0.53	0.003
Cognitive (CF)	73.89 (24.24)	75.55 (27.24)	0.06	0.431
Social (SF)	20.56 (25.40)	35.56 (33.82)	0.50	0.012
Symptoms/Single Items^b^				
Fatigue (FA)	68.33 (24.20)	56.29 (28.12)	0.45	0.022
Nausea (NV)	25.00 (28.28)	20.55 (24.24)	0.16	0.243
Pain (PA)	72.22 (33.99)	57.22 (34.35)	0.43	0.028
Dyspnoea (DY)	38.89 (39.22)	12.22 (28.34)	0.77	0.002
Insomnia (SL)	57.78 (66.67)	35.56 (36.04)	0.41	0.013
Appetite Loss (AP)	60.00 (39.53)	44.44 (36.40)	0.40	0.030
Constipation (CO)	32.22 (38.63)	20.00 (34.57)	0.33	0.004
Diarrhoea (DI)	21.11 (30.92)	10.00 (27.88)	0.37	0.097
Financial (FI)	78.89 (29.66)	65.55 (33.31)	0.41	0.009

^a^The higher score, the better the level of functioning

^b^The higher the score, the worse the symptoms/problems

*P* < 0.05 indicate significance

Table 3 shows that some subgroups had a significant effect on QoL. There was a significant difference related to the patient’s sex on global health status/QoL (*p* = 0.038), DY (*p* = 0.046), and CO (*p* = 0.030). The patient’s age significantly affected the scales of global health status/QoL (*p* = 0.002) and FI (*p* = 0.039). There were also significant effects of the caregivers’ age on DY (*p* = 0.031) and caregivers’ experience on DY (*p* = 0.030).

**Table 3:** Demographic variables that effect quality of life^a^

Variable	GH	EF	SF	FA	PA	DY	SL	AP	CO	FI
Sex patient^a^										
Male	0.038	-	-	-	-	0.046	-	-	0.030	-
Female										
Age patient^b^										
< 44 years old	0.002	-	-	-	-	-	-	-	-	0.039
45–54 years old										
> 55 years old										
Sex Family caregiver^a^										
Male	-	-	-	-	-	-	-	-	-	-
Female										
Age Family Caregiver^b^										
< 44 years old	-	-	-	-	-	0.031	-	-	-	-
45–54 years old										
> 55 years old										
Relationship with patient^a^										
Spouse	-	-	-	-	-	-	-	-	-	-
Non-spouse										
Educational background of family caregiver^b^										
Elementary	-	-	-	-	-	-	-	-	-	-
Junior high										
Senior high										
University										
Experience in caregiving of family caregiver^a^										
Yes	-	-	-	-	-	-	-	-	-	-
No										

^a^Paired t-test

^b^One-way ANOVA

*P* < 0.05 indicate significance

## Discussion

The aim of the current study is piloting and testing an intervention given to the family caregivers on the quality of life of palliative cancer patients. As indicated in the Results section, there were several items on the QoL scale that increased significantly after intervention, namely, global health status/QoL emotional and social functioning, as well as seven items on symptoms and single items, namely, fatigue, pain, dyspnea, insomnia, appetite loss, constipation, and financial issues.

A positive impact of intervention may result for several reasons. First, although McMillan and Weitzner (1998) suggested that the QoL of patients in terminal condition decreased mostly because of declining physical functioning, while their social and spiritual functioning were relatively high [17]. However, improved global health status/QoL of patients in the present study may relate to emotional and social aspects that were facilitated during the intervention.

Second, the substantial increase of emotional and social functioning may be attributable to the intervention being developed mostly to enhance family caregiver involvement in palliative patient care [18]. Being together with close friends and family members is possibly the most important coping strategy for individuals facing impending death from cancer [19]. Providing constructive social support is also proven beneficial in reducing pain and lowering the number of depressive symptoms [20]. As participants in the current study were mostly immobile, family caregivers might become the crucial source for social support [21]. Provision of basic nursing care likely provides the opportunity for social conversation, which helps keep the patient engaged in family life. Emotional support also can be enhanced by involving the patients as active participants, as this involvement helps maintain the patients’ dignity [22] and may enhance their personal sense of meaning through active engagement with palliative care providers [23, 24]. During the training, the nurse educators encouraged the family caregivers to involve the patients in their own care as much as possible.

Third, it is also possible that providing comfort measures, such as changing their positions and improving their hygiene, helps patients reduce focus on their physical discomfort and makes them more capable of staying socially and emotionally engaged [23]. From a palliative care perspective, most patients consider self-esteem and personal image to be important aspects. They want to stay physically clean and free from odors and bodily fluids, and want to have a normal appearance despite their being in the dying process [25].

In summary, most symptoms declined after an intervention given at a time when terminal patients tend to suffer a decline after hospital discharge. These improvements can be linked to the procedures provided in the intervention in the current study, such as showering, hair washing, feeding, assisting with toileting, physical repositioning, and oral hygiene. The latter is especially important in countering side effects of chemotherapy and radiation, such as dry mouth and decreased appetite [26].

A previous meta-analysis showed that interventions given to family caregivers of patients were divided into three types: psycho-education, skills training focused on coping and problem solving, and therapeutic counseling [27]. The study made no mention of skills training, which provides essential information for practical steps in handling routine daily living activities and personal care. One reason for this difference in protocols may be the meta-analysis’ being limited to developed countries. Therefore, interventions conducted in the current research are considered more appropriate for the context of this pilot study, where many terminal cancer patients live out their life at home with their family as a palliative care provider, as is very common in Indonesia and much of Asia.

Family caregivers in Asian countries generally stay with the patient constantly. They are usually willing to provide any type of palliative care necessary. When the patient is staying at home and lacks ability to complete daily living activities, most family caregivers are not equipped to handle the challenges involved in assisting them [27]. Asian patients may also feel more comfortable receiving help from their own relatives. The current study used an educational package consisting of an instruction manual and informational video, along with demonstrations by nurse educators. The video was added to provide visual demonstration and make the manual’s instructions easier to comprehend. Family caregivers can refer to the manual, which contains detailed explanation and pictures, when they need more information. Recent research on similar interventions using only manuals showed that health professionals used this as a complementary aid. Health professionals shared a variety of experiences in palliative care, and stated they were more comfortable using their own ways of providing information [28]. Therefore, an intervention’s success may vary depending on the innovations health professionals produce.

### Strengths and limitations

The current study measured the impact of family caregivers’ skills training on patients’ QoL, as there is a lack of evidence about specific nursing education interventions in this area [27], While limited by the small sample patient population in this pilot study (*n* = 30), the results are unique because there are few, if any, studies that directly address intervention protocols for the palliative care providers of terminal cancer patients from low- to medium-income countries [29]. However, a positive impact on QoL through palliative care has been demonstrated [30]. Our study was valuable because, to our knowledge, this was the first Indonesia-based study that attempted, by using an instructional module with a manual and supplemental video, to provide basic skills training for family caregivers of palliative cancer patients. The intervention also attempted to combine provision of an educational package with direct teaching sessions, and set up the follow-up at home to further encourage the family caregivers.

The main limitation of the present study was the absence of a control group. However, given the sensitive nature of the topic of death and dying, and the scarcity of research in the area of palliative care in Indonesia, a decision was made to delimit this initial study in order to assess acceptability of the outcome measures to this vulnerable population. Completion of the advance and follow-up questionnaires did not appear to cause any distress for the patients or their relatives. This opens the door for the next stage of investigation, which would be a randomized controlled trial of the intervention for palliative care with terminally ill cancer patients in Indonesia, or in other Southeast Asian countries with similar conditions.

## Conclusions

The present study provides essential data, which will serve as a basis for subsequent investigations of the provision of palliative care in developing countries. It is recommended that a larger-scale study with the addition of a control group be conducted to further delineate the benefits of provision of BST to family members of cancer patients. Because the interventions and teaching aids herein were simple and affordable, it is also recommended that similar studies in urban, suburban, and rural areas be conducted to investigate differences in acceptance of the program. Given that financial aspects are one of Indonesians’ main concerns, it is recommended that they also be taken into account in future research in an Indonesian setting. Finally, prior to any randomized control trial, it is suggested that psychometric adaptations are made to the EORTC QLQ C-30 to accommodate the economic and cultural sensitivities of terminally ill Indonesian patients, and to facilitate effective interpretation of the scores.

## Abbreviations

AP: Appetite loss
BST: Basic skills training
CF: Cognitive functioning
CO: Constipation
DI: Diarrhea
DY: Dyspnea
EF: Emotional functioning
EORTC QLQ C30: European Organization for Research and Treatment Cancer Quality of Life C30
FA: Fatigue
FI: Financial hardship related to illness
NV: Nausea/vomiting
PA: Pain
PF: Physical functioning
PPS: Palliative performance scale
QoL: Quality of life
RF: Role functioning
SF: Social functioning
SL: Insomnia/sleep
